# The art of medicine: COVID-19, online shaming, and health-care professionals

**DOI:** 10.1016/S0140-6736(21)01706-2

**Published:** 2021-08-07

**Authors:** Luna Dolezal, Arthur Rose, Fred Cooper

**Affiliations:** Wellcome Centre for Cultures and Environments of Health, University of Exeter, Exeter EX4 4QH, UK

Stigma and shame have been features of past pandemics. The stigma associated with disease can be experienced as shame by those who spread it. In almost all human cultures, there is shame attached to being “contaminated”, to the vulnerability inherent in illness, and to potentially spreading a disease to others. As previous pandemics have taught us, coming into contact with, or being associated with, a highly infectious and potentially deadly disease has social consequences. Hence, it is no surprise that stigma and shame have developed around COVID-19. Although there have been outpourings of support and admiration for health-care workers for their dedicated service in this pandemic, health professionals have also been among those directly affected by shaming practices.

In previous epidemics, health-care workers have been shunned, feared, and treated with suspicion. Doctors on HIV wards in the UK during the 1990s, for example, described how anxieties around “contamination” led colleagues in other specialties to stigmatise their work as devalued and shameful. In an outbreak of Ebola virus disease in Uganda in 2000-01, one nurse heartbreakingly recalled: “Our clothes were burned, and our children kept away from us, our families shunned us and were afraid of us.” Health-care workers on the front line of the COVID-19 response have been similarly shamed, sometimes leading to violence and abuse. The fear of contamination and of spreading SARS-CoV-2 has led to nurses in the UK being attacked on the street; care workers being verbally abused in supermarkets; and paramedics being evicted from their homes. Worldwide, doctors have been shamed and bullied for refusing to work without adequate personal protective equipment (PPE), while the wartime rhetoric deployed by some politicians set up pernicious expectations of personal sacrifice for health professionals.

**Figure:**
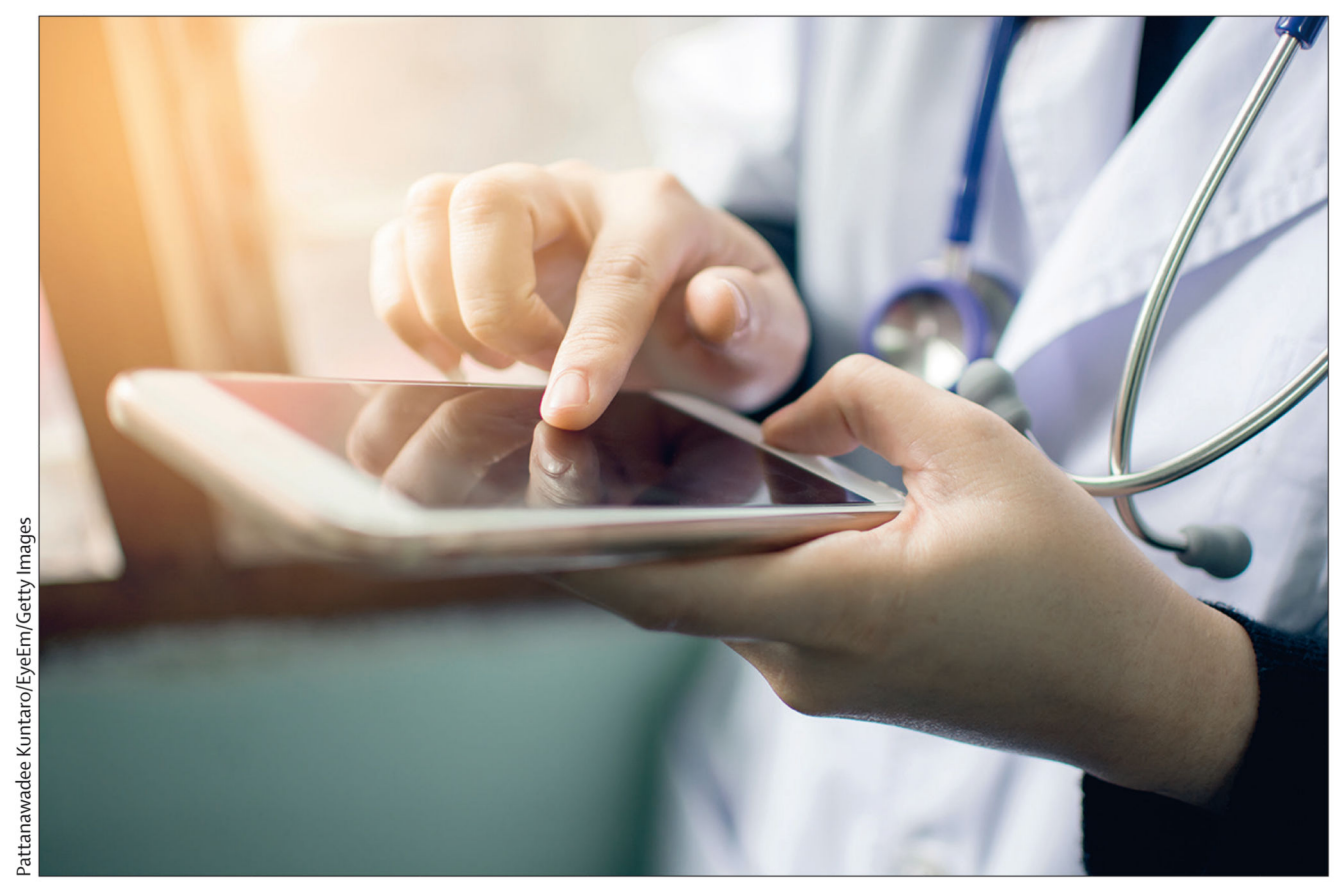
Pattanawadee Kuntaro/EyeEm/Getty Images

What seems to set COVID-19 apart from earlier events is the extent of shaming that has been fuelled by social media use. Online shaming and outrage in this pandemic have channelled fears and uncertainties about the disease and frustrations with disruptive public health measures put in place to control COVID-19. Unfortunately, doctors, along with other health-care workers, have become the targets of online shaming pile-ons.

Among those subjected to public shaming in 2020 were Chris Higgins, an Australian general practitioner, and Jean-Robert Ngola, a physician who worked at the time in New Brunswick, Canada. Higgins had continued to see his patients in his Melbourne practice while he had what he thought was a mild cold after a trip to the USA, before he tested positive for SARS-CoV-2. Ngola did not initially self-isolate on his return from crossing the border with Quebec (he said that the rules were not clear and he was following the same practices as people around him) and was accused of violating the New Brunswick Emergency Measures Act. Such cases occurred in the context of global medical cultures of overwork, in which health-care professionals may be expected to go to work when they are unwell and feel pressured if they take time off. Both physicians were singled out by politicians and although they were not mentioned by name, local news sources and social media accounts identified them, and they were subjected to online shaming. Ngola’s shaming was further inflected with racist vitriol. Charges against Ngola of breaching the provincial Emergency Measures Act were eventually withdrawn in 2021, but these events underscored his precarious status as a racialised doctor.

In the UK, as elsewhere, the online shaming directed at doctors has shifted its focus throughout the pandemic, from shaming health-care professionals for potentially spreading the virus to discrediting them as witnesses. Shortly before an appearance on the BBC’s television programme *Question Time* in January, 2021, the palliative care doctor and writer Rachel Clarke tweeted the day’s number of COVID-19 deaths, swiftly followed by this pre-emptive message: “And before the deluge of abuse begins, don’t bother. I am seeing these poor human beings in their final days, hours and moments of life. I am seeing it—day after day after day—and it’s utterly, horribly heartbreaking.” Clarke and other health professionals have been shamed, harassed, and threatened by COVID-19 sceptics who continue to allege that the virus is a hoax and that doctors are party to a deception to fool or mislead the public. The reasons for this shaming appear substantively different. Rather than targeting doctors for potentially spreading SARS-CoV-2 or for falling short of the expectations of inhuman infallibility routinely imposed on doctors, the shaming referred to in Clarke’s Tweet is directed towards what doctors are seeing, witnessing, and saying about the toll of COVID-19.

Public shaming used to take place in the public square, where members of one’s immediate community would mete out judgment and punishment for social and moral transgressions. From the public square, to newspapers, to television, nowadays public shaming has moved primarily to the internet. This move to the online arena is not merely a shift in the medium of shaming. It creates a whole new set of conditions for its occurrence. The internet, a domain with ample opportunity for anonymity, lacks the geography and temporality of previous sites of public shaming. As the philosopher Bonnie Mann has described in relation to online gender-based shaming, the “scene of shame” has changed dramatically with the internet. Online shame is unbounded by space and time, it can follow an individual everywhere and indefinitely. Shaming is transformed from something fleeting into a permanent record of a real or alleged mistake or transgression: a “digital scarlet letter”, as Dean Levmore and Martha Nussbaum describe it. In this way, online shaming can amplify a minor transgression into a major life-changing incident. It can generate vitriol, abuse, and hate for an entirely false accusation, leading nonetheless to reputational damage and personal harm.

While online shaming usually stems from two primary motivations, rectification (righting some perceived wrong) and cruelty (revenge, degradation, or an attack for personal reasons), these are by no means mutually exclusive. Online shaming that is used to target individuals for actions that are deemed “undesirable” or “unacceptable” can also involve a cruel attack in order to further personally “punish” an individual for a real or alleged transgression. These dynamics of rectification and cruelty online are increasingly intruding into the lives of some health-care professionals. A shaming response for a perceived mistake can rapidly be transformed into cruel attacks on one’s character, family, reputation, and livelihood, and on occasion with threats of violence and death.

In 2020, the resurgence of the hashtag #NoToDoctorShaming on Twitter highlighted the problem of online shaming of health professionals. Doctors are often held up to superhuman standards that demand infallibility. Common human occurrences, such as making mistakes, falling sick, needing sleep, or displaying emotion could be potential opportunities for shaming. Working on the front line of medicine, health-care workers are often blamed, or are perceived to be responsible, for system-wide problems such as staff shortages, short appointment times, insufficient bed space, PPE shortages, long waiting lists, or limited treatment options. Such pressures are compounded by the emotional and physical strain that comes with long working hours and having high caseloads, all of which have been exacerbated during the COVID-19 crisis.

Because online shaming is often unbounded by time, place, and scale, its effects are unpredictable: it may dissipate quickly with no ill effects; it may lead to a shaming backlash; or it may result in a shaming pile-on that has devastating and long-lasting effects. While shaming an individual can sometimes lead to positive change, it is more likely to have negative consequences and could lead to defensiveness, anxiety, social withdrawal, or adverse effects on one’s mental health. As testimonies by its victims suggest, online shaming may lead to unemployment, reputational damage, and compromised social and intimate relationships. It could also exacerbate cultures of shame that may exist in some health-care settings. Research has shown how health-care staff have reported feelings of fear and being blamed or shamed in their workplaces in relation to the reporting of medical error and highlighted the importance of moving away from a blame culture to a restorative culture of learning. Additionally, online shaming also highlights the broader shaming culture that has become commonplace in the public sphere.

Although some health professionals are the recipients of online shaming, others have used social media as a social and political tool. Australian general practitioners expressed their support for Higgins on social media after his online shaming. Clarke and others are using their voices on Twitter and in the media to, among other things, highlight poor public health policy and to call to account those responsible. Yet the unbounded nature of online shaming means that even such “good” uses of shaming may have unexpected impacts. The problem with online shaming is that its consequences are unpredictable. We can never be sure how it will land and what effects it will have.
